# Characteristic calcification behavior of five surgical aortic valve bioprostheses models: An in vitro study

**DOI:** 10.1002/hsr2.2304

**Published:** 2024-08-20

**Authors:** Najla Sadat, John H. Lojenburg, Michael Scharfschwerdt, Matthias Klinger, Buntaro Fujita, Stephan Ensminger

**Affiliations:** ^1^ Department of Cardiac and Thoracic Vascular Surgery University Medical Center Schleswig‐Holstein Lübeck Germany; ^2^ DZHK (German Center for Cardiovascular Research), Partner Site Hamburg/Kiel Lübeck Germany; ^3^ Institute for Anatomy, Medical Faculty University of Lübeck Lübeck Germany

**Keywords:** bioprostheses degeneration, calcification, durability testing, structural valve deterioration, surgical aortic valve

## Abstract

**Background and Aims:**

The durability of surgical aortic valve bioprostheses (SAV) is limited by the calcification of the leaflets, which results in degeneration. In clinical routine, there seems to be substantial variability in the degeneration of specific SAV models. Our study aims to establish an in vitro calcification model for prosthetic valves, characterizing the calcification behavior of different SAVs.

**Methods:**

Five commercially available SAV models (Epic™ Supra, Freestyle®, Intuity®, Perimount®, and Trifecta™) were perfused with double‐distilled water and physiological buffer with a defined calcium concentration (CaCl_2_ = 1.5 mM) at 37°C over 32.9 million cycles in a Hi‐Cycle tester which corresponds to approximately 1 patient‐year (calcified group). Untreated prosthetic valves served as the negative control group (noncalcified group). Calcium titration, scanning electron microscopy (SEM), histological examination, and tissue thickness measurements were performed to evaluate noncalcified and calcified SAVs (*n* = 10).

**Results:**

Treatment in the Hi‐Cycle tester with calcification buffer maintained significantly higher calcium absorption of SAVs compared to the control group (*p* < 0.001). Additionally, hydroxyapatite crystals were found in the calcified SAV in SEM. Porcine valves rarely demonstrated punctual calcification pattern, while bovine pericardial valves presented distinct planar calcification pattern in histological examination. Further, calcification content differed significantly depending on the SAV model, with the highest calcium content in Trifecta (213 µg/cm^2^) and the lowest in Epic Supra (8 µg/cm^2^) (*p* < 0.001).

**Conclusion:**

Data from our study revealed significant differences in leaflet calcification for the various aortic valve bioprostheses models. Further studies are necessary to investigate whether these findings can mimic the clinical data of the implanted prostheses.

## 1 INTRODUCTION

Bioprosthetic substitutes are classified as homografts, pulmonary autografts, stentless, and stented, or are divided according to the implantation technique in surgical or transcatheter valves. Further separation in porcine and pericardial bovine valves considers the biological material used for the leaflets.^1^ The contra‐partner of the bioprosthetic heart valves (BHVs) are mechanical valves with a clear superiority regarding durability compared to BHVs. Clinical selection between these two valve models and valve replacement are performed in accordance with the guidelines for the management of patients with valvular heart disease.^2^, ^3^ Additionally, the guidelines give recommendations for BHV—surgical valve versus transcatheter valve. But there is any recommendation regarding specific surgical valve models porcine vs bovine, or which specific surgical valve model should be implanted in which cases. The common advantage of all BHVs is avoiding lifelong anticoagulation. On the other side, structural valve degeneration remains the Achilles heel of BHVs, resulting in limited durability of these heart valves.^4^ In this regard, biological degeneration—mostly calcification—plays a main role, additionally to mechanical stress.^5^, ^6^

First, known predictors of structural valve deterioration (SVD) include patient‐related risk factors such as young age, renal failure, dialysis treatment, hyperparathyroidism, and other metabolic disorders such as hyperlipidaemia, causing early deterioration of BHVs.^7^ Second, prosthesis‐related factors such as tissue material—porcine versus bovine pericardial‐ and the valve design may also impact durability.^8^, ^9^ In this context, increasing numbers of BHVs demand systematically analyzes under standardized conditions for a better understanding of the various patterns of BHV calcification to prevent this process and consequently enhance the durability of BHVs. Therefore, the aim of this study was to investigate characteristic calcification patterns of different surgical aortic valve models in a standardized in vitro setting.

## 2 METHODS

### 2.1 Ethical statement

Patients and animals were not involved in this in vitro study.

### 2.2 Aortic valve bioprostheses

Our investigation includes five different SAV models with the labeled size 23 mm (total *n* = 10). We used Epic Supra (Abbott) as stented and Freestyle® (Medtronic) as stentless porcine valve models. As bovine pericardium valves, we choose the Perimount® (Edwards Lifesciences) and the externally mounted Trifecta™ GT (Abbott) for stented valves. In addition, the rapid deployment valve Intuity® (Edwards Lifesciences) was also investigated. Each BHV model was randomized between the noncalcified (control group; SAV without any treatment) and the calcified group (SAV were treated with calcification puffer).

### 2.3 Calcification‐puffer and durability testing

The physiological buffer with double‐distilled water, a defined calcium concentration of 1.5 mmol and a pH of 7.4 was modified as previously published and was used as a calcification solution for durability testing (Table 1).^10^, ^11^ Additionally, hydrochloric acid 0.05% (EMSURE® MERCK) and sodium hydroxide 0.01 M (EMSURE® MERCK) were used for pH adjustment. The calcification solution was prepared at room temperature. The durability testing was performed in a Hi‐Cycle tester (ViVitro Labs Inc.) according to ISO‐5840 standards under dynamic condition with a frequency of 500/min and a temperature of 37°C (Video S1).^12^, ^13^ This Hi‐Cycle model consists of six plexiglass compartments, which are connected to each other. This way, the buffer circulates inside the six compartments and constantly perfuses all bioprostheses. The buffer was changed weekly. Overall, the bioprostheses were under dynamic testing of over 32.921 million cycles in the Hi‐Cycle tester, which corresponds to approximately 1 patient‐year (calcified group). After durability testing, the leaflets were removed from the frame for analyzes of leaflet calcification. Also, the leaflets of the control group (noncalcified group) were prepared from the frame for further investigations.

**Table 1:** Buffer solution for durability testing.

	Concentration (mmol)
CaCl_2_ (calcium chloride dihydrate)	1.5
KCl (potassium chloride)	1.2
KH_2_PO_4_ (potassium dihydrogen phosphate)	1.25
Na_2_HPO_4_ (di‐sodium hydrogen phosphate)	0.32
NaH_2_PO_4_ (sodium dihydrogen phosphate)	0.11

### 2.4 Analysis of tissue thickness

The transmission light absorption method was used to transilluminate the leaflets with blue light (spectral response 470 nm) for detection of the tissue thickness with a software program (STOTM—soft tissue optical thickness measurement). Pictures were recorded by a high‐resolution camera (Canon EOS 300D) to analyze the calcification pattern. High tissue thickness is shown as white marks within the leaflets.

### 2.5 Histological and scanning electron microscopic (SEM) examination

Calcified leaflets of each SAV model were compared with noncalcified leaflets by histological and SEM examinations. For histological examination, the leaflets were fixed in paraffin and were cut into 6 µm tissue sections for staining. Histological staining (Carl Roth) was performed with von Kossa and Alizarin Red S for analyzes of biomineralization, while Haematoxylin & Eosin and elastic van Gieson were used for tissue evaluation. Additionally, valve tissue was analyzed for biomineralization by scanning electron microscope (Zeiss EVO, Carl Zeiss Microscopy GmbH) before and after calcification.

### 2.6 Calcium titration

Calcium titration was applied to extract calcium from the leaflets. First, the valves were shaken in 10 mL 0.1 M hydrochloric acid (EMSURE® MERCK) in a Cello shaker to extract the calcium from the valve for 30 min. A solution constainingof 88 mL double distilled water and 2 mL 25% ammoniac (Carl Roth), was added, and the valve was removed. One indicator buffer tablet (for the determination of water hardness with Titriplex solution; contains ammonium chloride, hexamethylenetetramine, Sigma‐Aldrich) was used to color the solution red for calcium detection. The 100 mL solution was separated into five 20 mL portions. Then, 0.001 M Ethylenediaminetetraacetic acid (EDTA; Sigma‐Aldrich) was titrated to the 20 mL red solution till the color changed to green. The defined content of EDTA was used to determine the content of calcium, as it is known that EDTA is linked 1:1 to calcium ions. Five titrations were performed for every untreated valve model (without calcification) and further five titrations for every calcified valve model.

### 2.7 Statistical analyzes

Data are presented as median and 25th–75th percentile. For statistical analyzes, SPSS for Mac (Version 29.0, IBM Corporation) was used. All variables were obtained from five measurements per prostheses model. A Shapiro–Wilk test was conducted to determine normality and the Mann–Whitney *U* test was used for comparison between two groups (noncalcified vs. calcified of the same valve model) as the data were nonnormal. Data evaluation was achieved through one‐way analysis of variance (ANOVA). A post hoc pairwise test was performed within several groups (valve models) using Tukey's adjustment for multiple testing. The tests were two‐sided. A *p*‐value of <0.05 was considered statistically significant.

## 3 RESULTS

### 3.1 Macroscopic evaluation and tissue thickness analysis

**Figure 1:**
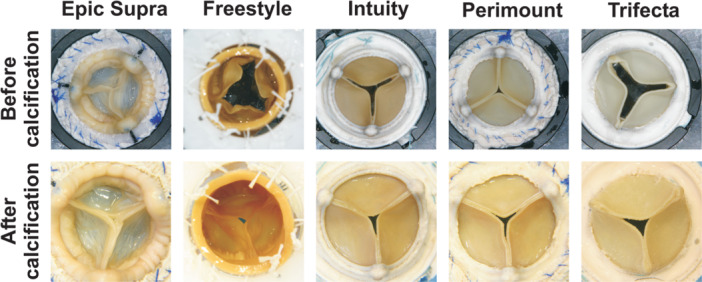
Surgical aortic valves before and after durability testing with calcification buffer.

**Figure 2:**
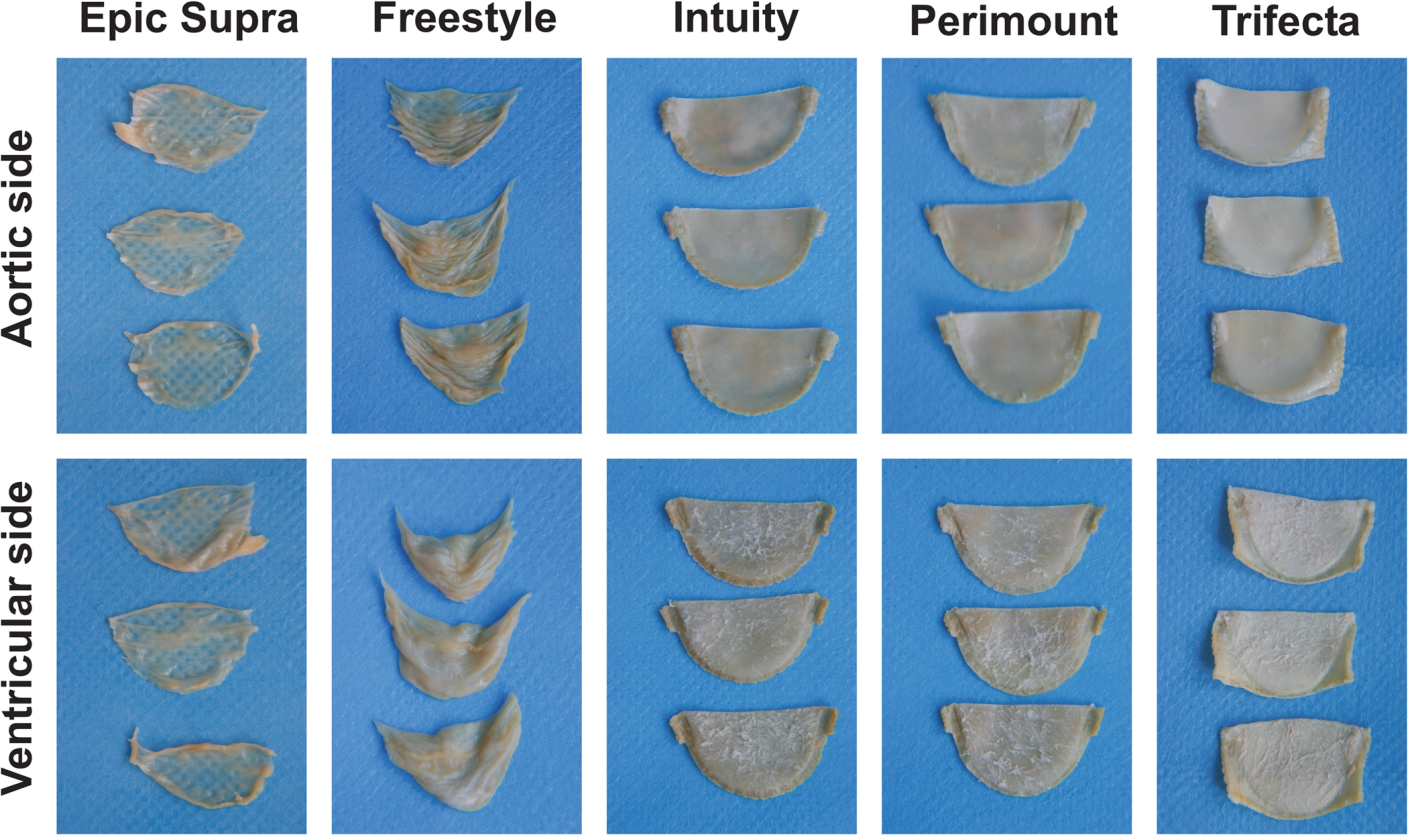
Macroscopic analyzes; leaflet calcification after durability testing; aortic side (inflow side) versus ventricular side (outflow side).

**Figure 3:**
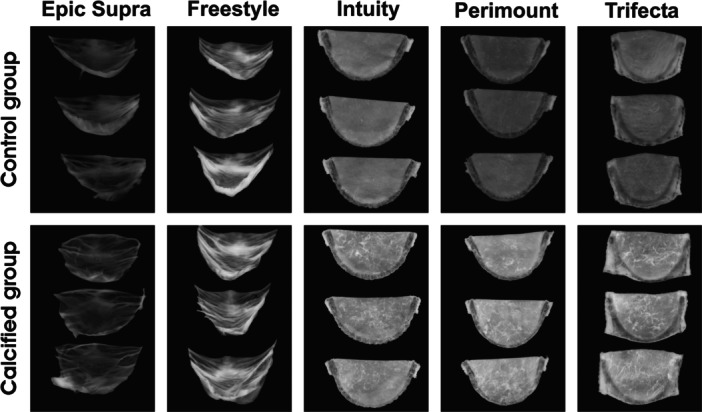
Analysis of tissue thickness; comparison of the control group (noncalcified) versus the calcified group. White marks present high tissue thickness within the valve leaflets.

Macroscopic examination presented distinct surface calcification of the Trifecta valve compared to Epic Supra and Freestyle (Figures 1 and 2). The macroscopically visible calcification increased from low to high in the following order: Epic Supra, Freestyle, Intuity, Perimount, and Trifecta. Further, the ventricular side of all valves was coarse compared to the control group and showed a higher manifestation of calcium foci than the aortic side of the same valve (Figure 2). Analyzes of tissue thickness demonstrated white reticular fibers as a sign of high tissue density—mostly located at the commissures and nadirs—as sign of calcification after the Hi‐Cycle procedure (Figure 3).

### 3.2 SEM

**Figure 4:**
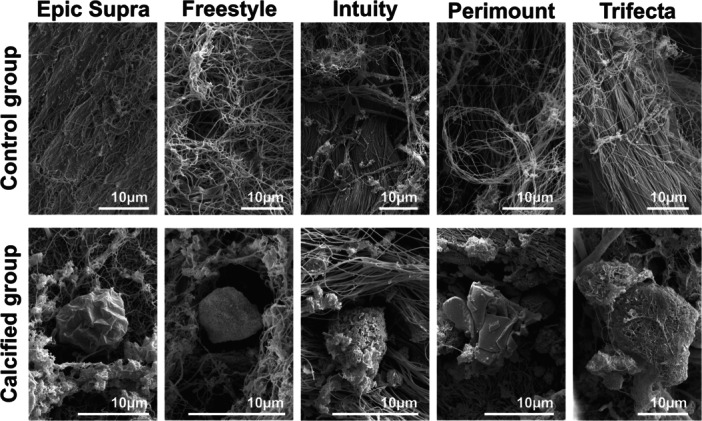
Scanning electron microscopy of the control group (noncalcified) and calcified valves; tissue structure in the noncalcified group; hydroxyapatite crystals in calcified valves; white bars represent 10 µm length.

Hydroxyapatite crystals were not found inside the control group, while SEM presented hydroxyapatite crystals as a proof of biomineralization after calcification treatment (calcified group) (Figure 4). The dimension and pattern of the hydroxyapatite crystals located inside the calcified group differed regarding the valve models. The Epic Supra and Freestyle prostheses as porcine pericardial tissue showed less and minor hydroxyapatite crystals, while the bovine pericardial valves demonstrated numerous major hydroxyapatite crystals (Figure 4). The hydroxyapatite crystal pattern was most intense inside the Trifecta after calcification treatment.

### 3.3 Histological examination

**Figure 5:**
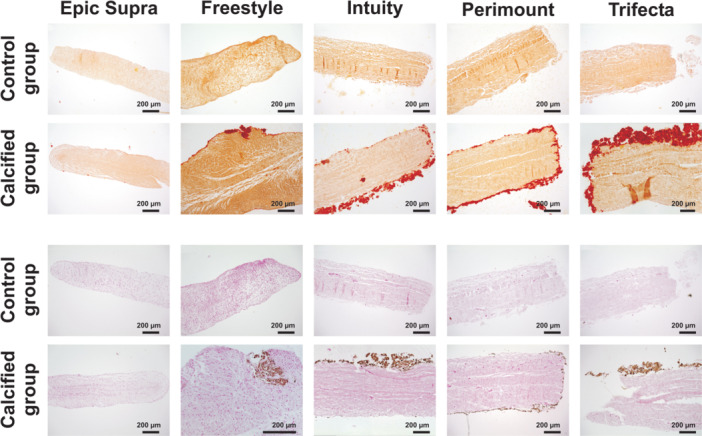
Histological examination I: of the noncalcified (control group) and the calcified surgical aortic valves; calcification painted red in Alizarin Red S and black in von Kossa staining.

**Figure 6:**
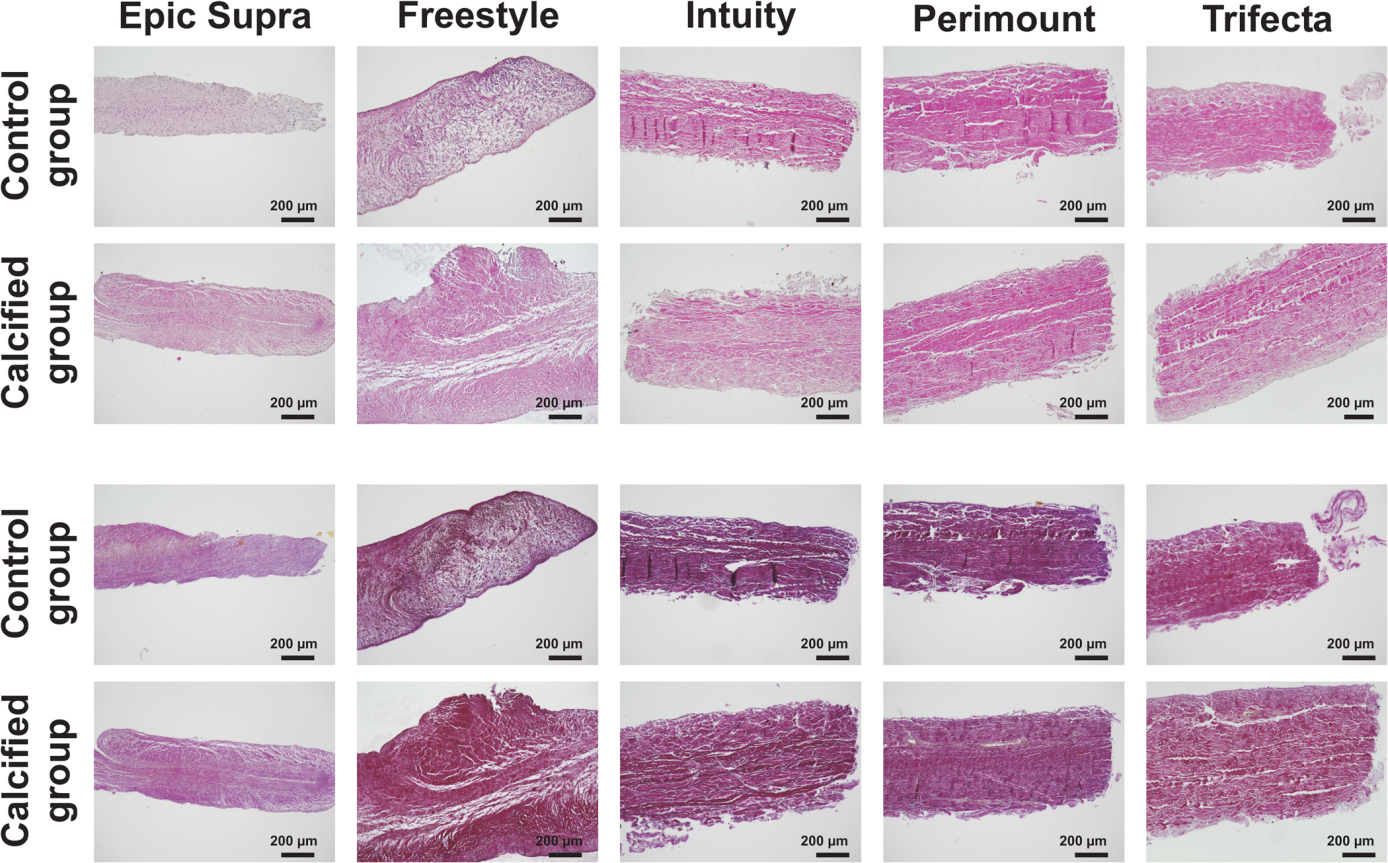
Histological examination II: of the noncalcified (control group) and the calcified surgical aortic valve tissue with Haematoxylin & Eosin and Elastica van Gieson staining.

Histological examination with Alizarin red and von Kossa staining clearly showed calcification of all the valves after durability testing with calcification buffer. In contrast, no calcification was detected in the control group (SAVs without treatment). After durability testing with calcium, the porcine valves Epic Supra and Freestyle contained small isolated punctual calcium nodules in Alizarin Red S staining. On the other side, the bovine pericardial valves showed a planar calcification pattern (Figures 5, S1, and S2). Trifecta showed the most pronounced calcification patterns in Alizarin red staining. Furthermore, endothelial damage, tissue decentralization, and loss of nucleus were noticed in Haematoxylin & Eosin staining after durability testing compared to the control group (Figures 6 and S3). Additionally, elastica van Gieson staining presented a loss of elastic fibers in all SAVs after treatment with calcification buffer (Figures 6 and S4). SVD was represented with a distinct tendency inside bovine pericardial valves versus porcine valves after calcification (Figures 5, 6, and S1–S4).

### 3.4 Calcium content

Calcium titration of the non‐calcified leaflets (control group) presented calcium content in a range of 2–4 µg/cm^2^ (Table 2). Durability testing with buffer lead to a significant increased calcium content within the same bioprostheses model (control group vs. calcified group): Epic Supra (3 vs. 8 µg/cm^2^; *p* < 0.001), Freestyle (2 vs. 17 µg/cm^2^; *p* < 0.001), Intuity (4 vs. 132 µg/cm^2^; *p* < 0.001), Perimount (3 vs. 179 µg/cm^2^; *p* < 0.001), and Trifecta (3 vs. 213 µg/cm^2^; *p* < 0.001) (Table 2). Additionally, calcium content differed significantly among calcified leaflets depending on the BHV model (Table 3). The porcine bioprostheses Epic Supra (8 µg/cm^2^) and Freestyle (17 µg/cm^2^) showed significantly lower calcium content versus bovine bioprostheses Intuity (132 µg/cm^2^), Perimount (179 µg/cm^2^), and Trifecta (213 µg/cm^2^) (Central Image and Table 2). The lowest calcium content was presented in Epic Supra (8 µg/cm^2^), while the highest calcium content was noticed in Trifecta (213 µg/cm^2^) after durability testing with calcification buffer (Central Image and Table 3). Comparison of the bovine pericardial valves revealed that Trifecta showed the highest calcium content (213 µg/cm^2^ valve area corresponds to 100%), Perimount (179 µg/cm^2^) presented with 16% lower, and Intuity (132 µg/cm^2^) with 38% lower calcium content after calcification treatment. Extreme differences were noticed between the bovine Trifecta versus the porcine Freestyle (17 µg/cm^2^) with 92% lower calcium content and the porcine Epic Supra (8 µg/cm^2^) with 96% lower calcium content.

**Figure:**
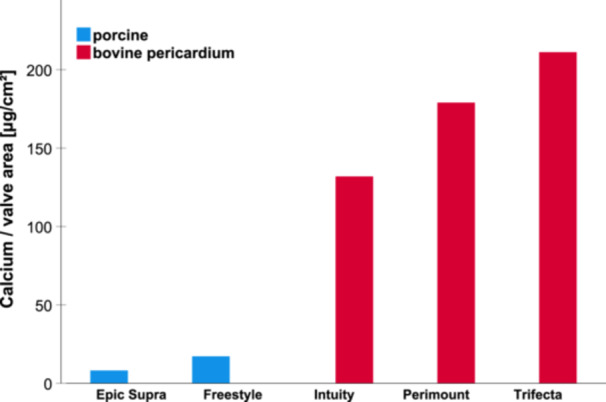
**Central Image:** Calcium content of the surgical aortic valve leaflets after treatment with calcium in the Hi‐Cyle tester.

**Table 2:** Calcium content of noncalcified versus calcified surgical aortic valve leaflets.

Surgical aortic valve	Noncalcified calcium (µg/cm^2^) median (25th–75th)	Calcified calcium (µg/cm^2^) median (25th–75th)	*p*‐Value
Epic Supra	3.209 (3.031–3.387)	7.724 (6.621–8.828)	<0.001
Freestyle	1.75 (1.313–2.188)	17.21 (17.122–17.21)	<0.001
Intuity	3.619 (3.429–3.619)	131.644 (130.854–132.434)	<0.001
Perimount	2.649 (2.649–2.89)	179.333 (178.763–179.618)	<0.001
Trifecta	2.56 (2.56–3.108)	213.412 (212.953–213.719)	<0.001

*Note*: Calcium content as median and 25th –75th percentile of five titrations per prostheses model.

**Table 3:** All pairwise‐comparison of calcified surgical aortic valves (SAV).

SAV	Calcium (µg/cm^2^) median (25th –75th)	All pairwise‐comparison (*p*‐value)			
		Epic Supra	Freestyle	Intuity	Perimount
Epic Supra	7.724 (6.621–8.828)				
Freestyle	17.21 (17.122–17.21)	<0.001			
Intuity	131.644 (130.854–132.434)	<0.001	<0.001		
Perimount	179.333 (178.763–179.618)	<0.001	<0.001	<0.001	
Trifecta	213.412 (212.953–213.719)	<0.001	<0.001	<0.001	<0.001

*Note*: Calcium content as median and 25th–75th percentile of five titration per prostheses model.

## 4 DISCUSSION

In this study, we present in vitro calcification patterns of five different SAV models, currently implanted in clinical settings. The main findings of this study can be summarized as follows:

1. Durability testing with calcification buffer induced significantly higher calcification content of the SAVs compared to the control group.
2. The calcification pattern differed depending on valve material, while isolated punctual calcification was found in porcine valves, the bovine pericardial valves demonstrated planar calcification pattern.
3. Specific valve models with the same tissue material showed significantly different calcification manifestations.
4. The lowest calcium content was found in the porcine Epic Supra and the highest in the bovine Trifecta.

### 4.1 Calcification mechanism

Degeneration of tissue valves is mainly caused by calcification resulting in limited durability when compared to mechanical valves. Restricting the tissue with a loss of elastic fibers and nucleus ‐ as detected in our histological examination—can accelerate calcification and structural valve degeneration (Figure 6). In this context, tissue alteration of BHVs may stimulate the pathophysiology of BHVs calcification in a similar way as native aortic valve calcification.^5^, ^14^ Usually, physiological cytoplasm‐calcium concentration is kept lower than extracellular calcium concentration by actively shifting calcium out of the healthy cell. But glutaraldehyde pretreatment of bioprosthetic valves to improve tissue durability, unfortunately, causes a loss of its physiological function by devitalization of cells. For this reason, calcification is accelerated within the devitalized residual cells of valve tissue by calcium accumulation from calcium‐containing extracellular fluid with membrane‐phosphorus.^7^ Here, the origin of calcium‐crystals rises and forms hydroxyapatite crystals consisting of calcium phosphate, the main mineral constituent of bone tissue. The shape and age of hydroxyapatite crystals can be recognized by characteristic marks and allow identification of calcification pattern.^15^, ^16^ Several in vitro models have been described previously including the Kiesendahl's et al. in vitro calcification model, where the feasibility of in vitro calcification of bioprosthetic material was reported. The authors used very high calcium concentration—partly out of the physiological range—in a pulse duplicator system calcifying bioprosthetic material (pericardiac patches or old valve models).^10^, ^11^, ^17^ Hence, too a high calcium concentration can lead to a false high calcium manifestation. Therefore, we preferred physiological calcium concentration (CaCl 1.5 mmol) in our model to mimic the in vivo conditions as closly as possible. Furthermore, the evidence of hydroxyapatite crystals was not proven in these in vitro studies. In our calcification model, we found specific hydroxyapatite crystals inside the valves after durability testing with a calcification buffer. Moreover, our new findings revealed a characteristic shape and quantity of hydroxyapatite, which differed with regard to the specific SAV model. The porcine valves were characterized by distinct small and lower hydroxyapatite crystals than bovine pericardial valves (e.g., Freestyle vs. Trifecta) (Figure 4). The calcification pattern was also confirmed in the histological examination with small isolated spotty calcium nodules in porcine valves and a prominent mainly planar calcification pattern in bovine pericardial valves (Figure 5). Furthermore, mechanical stress was simulated by high‐frequency durability testing in our in vitro model and demonstrated more tissue alteration and calcification on the ventricular side of the leaflets as compared to the aortic side. Also, areas of highest shear stress—the commissures and nadirs of the leaflets—showed pronounced calcification (Figures 2 and 3). Interestingly, these differences have become noticeable already after simulating 1 year of calcification treatment. A further possible reason for the early degeneration of BHVs could be the glutaraldehyde pretreatment of BHVs, as this destroys the residual cell and cell fragments in bioprosthetic tissue by chemical cross‐linking.^5^, ^7^ In addition, the different valve models were pretreated with various anti‐calcification manufacture‐protocols where the concentration of glutaraldehyde can also have a variable impact on calcification pattern in BHVs. While 0.6% or less glutaraldehyde seems to initiate calcification, 3% glutaraldehyde is paradoxically described as an anti‐calcification substance.^18^ Cross‐linking effect and cytotoxicity of low glutaraldehyde concentration seem to be possible explanations for this paradox effect.^19^ However, the detailed mechanism is still not entirely understood. Hence, manufacturers nonuniform anti‐calcification treatment protocols may cause several characteristic calcification behaviors within the same material, in addition to different patterns of mechanical stress inside the valve, obviously depending on the valve design.

### 4.2 Impact of valve material and design on calcification behavior

Our results with pericardial bovine valves revealed that Trifecta absorbed about 16% more calcium content versus Intuity and 38% more calcium content versus Perimount. Within the porcine valves, the difference in calcium content was nearly twice as high—Freestyle versus Epic Supra (17 vs. 8 µg/cm^2^ valve area). Consequently, this variety in calcium absorption could explain the different durability and degeneration pattern for the respective BHVs. Vogt et al. reported in a clinical analysis (German aortic valve register; GARY data) that early degeneration of the Trifecta valve, in accordance with our in vitro data, with significant higher redo or reintervention compared to Perimount valves and porcine valves (Epic and Hancock II) notable even 5 years after implantation.^20^ Additionally, a comparison of the Mosaic Ultra (porcine valve) and the Perimount Magna (bovine pericardial valve) underlines our investigation and demonstrated faster and more severe in vitro calcification of the Magna valve versus. the Mosaic Ultra.^17^ On the one side, porcine valves showed less calcification tendency in clinical experience, but on the other side, porcine valves display more frequently leaflet tears as compared to bovine pericardial valves after long‐term clinical evaluation.^9^, ^21^ Our analyzes presented significant differences even within the valves with the same tissue material—for example, Trifecta versus Perimount versus intuity. However, clinical comparison of porcine versus bovine without further specification should be critically evaluated with regard to valve models and design. The Hancock II, Mosaic Ultra, Epic Supra, and Freestyle—as porcine valves—seem to show low calcification rates in comparison to Perimount Magna, Perimount, and Intuity as—bovine pericardial valves with moderate calcification behavior. Interestingly, Trifecta—also a bovine pericardial valve—seems to have an extreme calcification tendency. A possible reason for this characteristic calcification behavior could be the externally mounted design of the valve causing more leaflet stress with leaflet alteration resulting in shorter durability.^22^ The leaflets of the Trifecta envelop the fairly rigid stent (Titanium) from the outside and are, therefore, exposed to higher mechanical stress. This may, in part, be an explanation for a recent finding in an analysis of the GARY, where there was a significant difference in risk for reoperation for various biological valves and, the Trifecta was associated with a reoperation rate >10% at 5‐year follow‐up.^20^ In contrast, for internally mounted valve designs, the leaflets are mounted inside the stent and the three commissures partially absorbing mechanical stress, in particular if the stent design is flexible. A further in vitro investigation of the hemodynamic properties of the Avalus and the Magna Ease (both with internally mounted leaflets) versus the Trifecta valve supported our observation.^23^ These discrepancies of surgical valves regarding durability required a recommendation of specific surgical valve model selection for special cases with high‐risk for calcification—such as renal failure, dialysis treatment, hyperparathyroidism, and other metabolic disorders—within the guidelines for the management of patients with valvular heart disease.^2^, ^3^ However, our in vitro model is promising and enables to analyze specific valve models regarding their calcification potential. Clinically relevant questions such as anti‐calcification treatment‐ can be investigated in a timely and cost‐efficient manner under standardized in vitro conditions to improve BHV durability with the intention of reducing or even preventing SVD. BHV implantation is shifting to younger patients, who have longer life expectancy but, unfortunately, a higher calcification potential resulting in early SVD. Recent studies reported that over 55 years, the outcome is similar with BHV and mechanical valves.^24^ But nowadays, a life expectancy of 85–90 years demands theoretically 30–35 years of BHV durability for implantation at the age of 55. This means the current average BHV durability of 10–15 years is about 20 years short of what is ideally needed. To close this gap, nowadays valve‐in‐valve procedures are performed and provide an alternative to redo‐surgery.^25^ However, this innovative recent procedure has also shown significant drawbacks such as patient‐prosthesis‐mismatch, paravalvular leakage, and thromboembolic events requiring anticoagulation. In particular, valve durability is essential when contemplating aortic valve replacement in patients with a long‐life expectancy. Our data showed the calcification of BHVs can already begin 1 year after implantation. At this early stage (stage 1 by Salaun et al. definition of bioprosthetic degeneration), calcification of the valve may not be clinically or hemodynamic relevant,^6^, ^17^ but an early calcification behavior of SAV models can impact valve durability and result in early SVD. Therefore, the selection of the primary SAV bioprostheses model is of utmost importance, and one should regard durability as essential for a long patient life expectancy and also risk factors for tissue valve calcification to achieve a best possible long‐term outcome.

## 5 STUDY LIMITATIONS

This study was performed with a simplified physiological fluid and does not include blood surface interaction and other blood components such as lipids, statins, proteins and immune cells, which also can contribute to calcification. Therefore, further studies are required to investigate the interaction of these components to discover drugs as anti‐calcification treatments to reduce or even prevent structural valve degeneration. Additionally, our in vitro study included only two SAVs per model. Further, prolonged durability testing is necessary to elucidate if these results can be confirmed and influence the long‐term outcome of patients.

## 6 CONCLUSION

Calcification patterns of BHVs differ significantly between different SAV models with superiority of porcine versus bovine pericardial valves under standardized conditions. The model‐specific calcification behavior of BHVs can be used for an individual and personalized selection approach for the right SAV model to enhance the durability of the valves, particularly in patients at high risk for calcification.

## AUTHOR CONTRIBUTIONS

**Najla Sadat**: Conceptualization; data curation; formal analysis; investigation; methodology; project administration; supervision; validation; visualization; writing—original draft; writing—review and editing. **John H. Lojenburg**: Data curation; visualization. **Michael Scharfschwerdt**: Methodology; software; supervision. **Matthias Klinger**: Methodology; resources; supervision; validation. **Buntaro Fujita**: Validation; writing—review and editing. **Stephan Ensminger**: Resources; validation; writing—review and editing.

## CONFLICT OF INTEREST STATEMENT

The authors declare no conflict of interest.

## TRANSPARENCY STATEMENT

The lead author Najla Sadat affirms that this manuscript is an honest, accurate, and transparent account of the study being reported; that no important aspects of the study have been omitted; and that any discrepancies from the study as planned (and, if relevant, registered) have been explained.

## Supporting information

Supporting information.

Supporting information.

Supporting information.

Supporting information.

Video 1: Surgical aortic valves during durability testing with calcium buffer.

## Data Availability

The data that support the findings of this study are available from the corresponding author upon reasonable request.
