# Supplementary material for: Characteristic calcification behavior of five surgical aortic valve bioprostheses models: An in vitro study
Source: Health Sci Rep. 2024 Aug 20;7(8):e2304. doi: 10.1002/hsr2.2304 (PMC11333945; doi:10.1002/hsr2.2304)
Supplement: Supplementary file 4 — Supporting information. [file HSR2-7-e2304-s004.pdf]

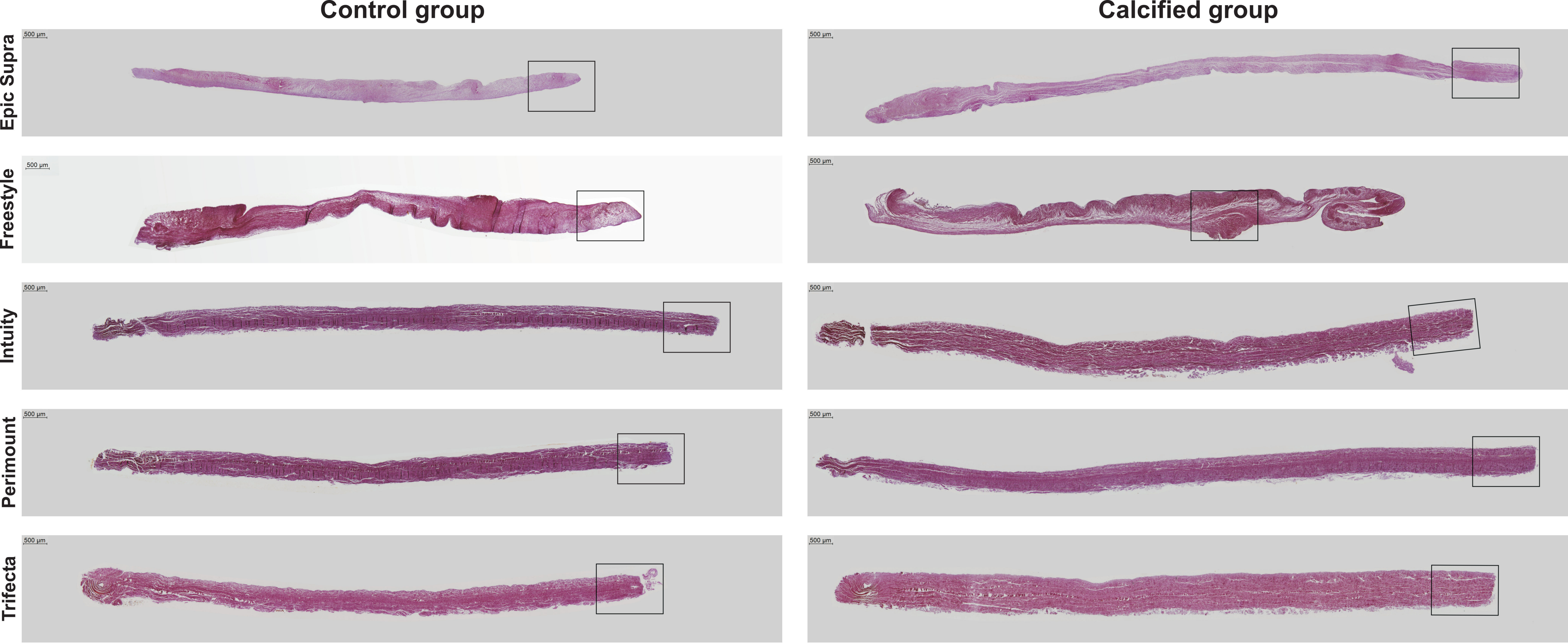

Figure S4: Histological examination; Elastica van Giessen staining of non-calcified (control group) and calcified valves
